# Geodesic Distance Algorithm for Extracting the Ascending Aorta from 3D CT Images

**DOI:** 10.1155/2016/4561979

**Published:** 2016-01-20

**Authors:** Yeonggul Jang, Ho Yub Jung, Youngtaek Hong, Iksung Cho, Hackjoon Shim, Hyuk-Jae Chang

**Affiliations:** ^1^Brain Korea 21 Project for Medical Science, Yonsei University, Seoul 120-752, Republic of Korea; ^2^Division of Computer and Electronic Systems Engineering, Hankuk University of Foreign Studies, Yongin 449-791, Republic of Korea; ^3^Division of Cardiology, Department of Internal Medicine, Severance Cardiovascular Hospital, Yonsei University College of Medicine, Seoul 120-752, Republic of Korea; ^4^Cardiovascular Research Institute, Yonsei University College of Medicine, Seoul 120-752, Republic of Korea

## Abstract

This paper presents a method for the automatic 3D segmentation of the ascending aorta from coronary computed tomography angiography (CCTA). The segmentation is performed in three steps. First, the initial seed points are selected by minimizing a newly proposed energy function across the Hough circles. Second, the ascending aorta is segmented by geodesic distance transformation. Third, the seed points are effectively transferred through the next axial slice by a novel transfer function. Experiments are performed using a database composed of 10 patients' CCTA images. For the experiment, the ground truths are annotated manually on the axial image slices by a medical expert. A comparative evaluation with state-of-the-art commercial aorta segmentation algorithms shows that our approach is computationally more efficient and accurate under the DSC (Dice Similarity Coefficient) measurements.

## 1. Introduction

As many risk factors, such as obesity and hypertension, are caused by irregular eating habits and the increased intake of fatty foods, the prevalence rate for cardiovascular diseases (CVDs) has also incrementally increased.

The recent rapid development of imaging techniques, such as computed tomography (CT) and magnetic resonance imaging (MRI), has made it possible to acquire an image of the moving heart that can be used to diagnose diverse CVDs. In particular, contrast-enhanced coronary CT angiography (CCTA) has become the premier modality for diagnosing CVDs with noninvasive visualization of the coronary arteries [1].

Aorta segmentation from the CCTA image is important for guiding coronary artery segmentation, as well as for the diagnosis of aortic diseases, such as aortic valve failure [2–4]. Aorta segmentation can be performed under two different conditions. First, under professional supervision, an object, such as the aorta in a medical image, is segmented using the input annotation of a professional user [5–7]. Second, the aorta is automatically detected and segmented without annotation interactions from a professional user [8–12]. For segmentation in most clinical routines for angiography, manual operation and annotation are still heavily relied upon for better accuracy under human supervision [2, 5, 7].

Although the results of manual segmentation are the most accurate, automatic aorta segmentation still has practical advantages over the manual segmentation approach. Obviously, the removal of human interaction is the main convenience because annotating the segmentation scribbles in 3D scans is often a very exacting process. Additionally, manual segmentation tends to vary between human experts and reproducibility can be a demanding factor for long-term medical image analysis. Ideally, an automatic aorta segmentation approach that has high accuracy similar to manual segmentation is preferred. Automatic and accurate aorta segmentation is still a very challenging problem but has the potential to assist in clinical routines for individualized cardiac risk stratification and therapy monitoring and to relieve human experts of difficult, tedious, and time consuming manual segmentation.

Previously, use of the automatic aorta segmentation method on a thoracic CT scan image was proposed by Kurugol et al. [3]. They used a Hough transform to detect the circles for the aorta and obtained the aorta centerline and radius functions through least squares spline fitting. The level set algorithm is used to evolve the initial boundary to the edge location, which required heavy computation time. Similarly, a method presented by Hennemuth et al. detects the ascending aorta with a Hough transform and performs segmentation by level sets [6]. However, Hennemuths method first requires manual removal of the rib area by human interaction, and the same large computational burden is present for the level set operation. Subasic et al. introduced another 3D level set deformable model to the aorta extraction problem [8]. They introduced a number of modifications to the original level set algorithm to reduce the computation time. However, incremental propagation of the level set was still slow. Additionally, their approach required minimal user interaction and was not a fully automated segmentation. Wang and Smedby used dynamic programming to detect the aortic boundary and estimate the aortic location [13]. To reduce computation time, the volume of interest (VOI) of the ascending aorta was used by Wang et al. [14]. First, they located the VOI based on the percentages of the image sizes and the gradients in the *X* and *Y* directions before detecting the location of the aorta. However, it is unsuitable for images with a various size of field of view (FOV) because it depends on the percentage of the image sizes.

In this paper, we present the fully automatic and fast segmentation method of the ascending aorta from the CCTA image using a geodesic distance algorithm combined with a Hough transform. The proposed algorithm has three steps with contributions in each part. The first step is to detect the aortic circle automatically and annotate the initial marking for segmentation. For efficient aorta circle detection, we adapt similar ideas from paper of Kurugol et al. [3]. However, we introduce new and efficient criteria for selecting the aortic circles among the multiple Hough circles detected by a 3D scan. Once the representative location of the aorta circle is found, the VOI is designated based on the center and radius of the aorta circle to reduce the computation time. Segmentation seed markings are chosen based on the aorta circle. In the second step, the ascending aorta is automatically segmented from the initial seed markings using a geodesic distance algorithm. In contrast to Subasic et al. [8] and Kurugol et al. [3], we tried to overcome the large computation time and obtain an accurate segmentation result using a fast geodesic distance algorithm without a 3D level set approach. Third, we introduce the seed transfer method to axial slices of the CCTA, which allows for the accurate segmentation on the adjacent axial slice. The proposed method is evaluated on 10 CCTA images and compared with ground truth segmentations, which were manually labeled by an experienced cardiologist using commercially available supervised segmentation programs. The accuracy measurement comparison is conducted using the conventional Dice Similarity Coefficient (DSC).

The paper is organized as follows: in Section 2, we will describe the proposed method in detail. In Section 3, we will present the evaluation results. In Section 4, we will discuss the proposed method. In Section 5, we will summarize the method with conclusions.

## 2. Methods

In this section, the proposed method is described in detail. The method first finds the most probable ascending aorta circle among the axial-slices. The standard circular Hough transform is used to generate possible ascending aorta candidates for the initial segmentation seeding. After the most probable ascending aorta circle is found, VOI is set up to improve the computation times. The ascending aorta is then segmented by the geodesic distance algorithm in VOI over the axial slices. The first segmented slice becomes the basis for segmentation seeding for the adjacent slices. The ascending aorta is segmented both upward and downward from the first slice until the aortic arch and aortic valve are reached, respectively. Instead of segmenting in a 3D space directly, the proposed approach accumulates and conjoins 2D segmentation results of axial slices. For relatively obtuse objects, such as the ascending aorta, a complex and time consuming 3D voxel segmentation is not necessary. The sequential appending of axial slice segmentations provides a fast and reliable separation of the ascending aorta by segmentation seed transfer.

### 2.1. Initial Aortic Circle Detection

The circular Hough transformation has been frequently used to detect the aorta. In this paper, we maintain the basic premise of the work of Kurugol et al. [3]. However, we also introduce a new approach for initializing the ascending aorta from the multiple Hough circle detections.

The Hough circle detections can lead to ambiguous results with an assorted number of circles. Various CT conditions can affect the detection of the Hough circles such as the intensity range and size of the ascending aorta. Additionally, various artifacts like beam hardening and step artifact may be included according to CCTA scan protocols such as tube voltage, reconstruction algorithm and filed of view (FOV), and detector size. Furthermore, the axial slices of angiography contain a diverse array of circular shapes that contribute to false positives. Various cues for selecting the aorta circle from miscellaneous Hough circles have been previously implemented. The relative position with respect to the trachea carina is a simple filtering criterion for the ascending aorta [3]. The intensity range of contrast agent is also an important clue for the selection. The Hough circles that are above and below the maximum and minimum radii are eliminated as candidates.

In this paper, we select the most probable ascending aorta circle for each axial slice of the scan. First, two Hough circles corresponding to the ascending and descending aorta are selected. Ascending and descending aortas have the same intensity range and a similar radius size range. Among the Hough circles that have a radius between the maximum and minimum lengths, two circles with the highest ratio of correct intensity ranges are selected as the aorta circles. In the second step, the ascending aorta circle is chosen between two selected Hough circles. The ascending aorta circle is expected to have a longer radius than the descending circle. The ascending aortas relative position in the axial-slice is also taken into consideration.

The axial slices that contain both ascending and descending aortic circles are below the aortic arch and above the left ventricle. Thus, we can expect to only find consistent aortic circles within the axial frames that contain both aorta circles. In other axial frames, the two detected aortic circles will not maintain their consistent position and size across adjacent frames. A reliable ascending aortic circle is selected from the axial frames by finding the slice that minimizes the energy function.

(1) Cm=argminCk⁡∑i=k−5k+5⌀Ci−⌀Ck+Cix,y−Ckx,y.

*C*_*i*_ and *C*_*k*_ are the ascending aortic circles that are found in slices *i* and *k*, respectively. *⌀* is the diameter of the circle. *C*_*i*_(*x*, *y*) is the center position of the circle. *C*_*m*_ represents the ascending aortic circle that remains most steady across the 11 axial slices. The 11 axial slices are chosen based on the tradeoff between the computation time and accuracy. The increased number of slices may be able to eliminate false positives better; however it also requires a larger computation time. Total of 11 axial slices, consisting of 5 slices up and 5 slices down from the middle slice, achieved a suitable balance between the accuracy and computation time. Increasing the slice number beyond 11 did not particularly make the aorta detection more accurate.

### 2.2. Volume of Interest (VOI)

Once the position of the ascending aorta is found by initial aortic circle detection, the VOI is constructed based on the center and radius of the detected circle. Because the aorta consists of a small portion of the scan image, limiting the computation range with VOI is a typical but effective method of reducing the computation time. Though the shape of an ascending aorta is largely aligned with the *z*-axis, an ascending aorta also slants in *x*-axis as it reaches the chamber. Therefore, a reasonably wide VOI range has been chosen. The *z*-range of VOI is between 0 and the *z*-axis size, and the starting and ending coordinates of VOI in the *X* and *Y* direction are determined as follows: *X*_1_ = max (*C*_*m*_(*x*) − 3 × *⌀C*_*m*_, 0);  *X*_2_ = min (*C*_*m*_(*x*) + 3 × *⌀C*_*m*_, *x*_size);  *Y*_1_ = max (*C*_*m*_(*y*) − 3 × *⌀C*_*m*_, 0); and *Y*_2_ = min (*C*_*m*_(*y*) − 3 × *⌀C*_*m*_, *y*_size), where *C*_*m*_(*x*) and *C*_*m*_(*y*) are the *x* and *y* positions of the center of the detected ascending aortic circle.

The detected aortic circle is padded by minimum and maximum *x* and *y* ranges that are 3 diameters away from the center of the aortic circle. Considering the consistent orientation of the ascending aorta during scanning, the rest of the undetected aortic voxels are expected to be found within the VOI range without complications.

### 2.3. Segmentation on Axial Slice

**Figure 1:**
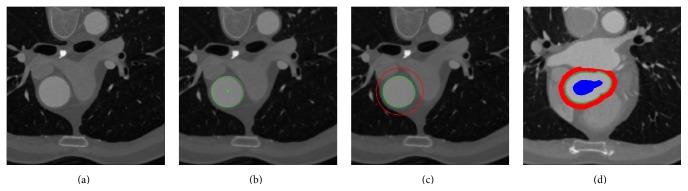
Detection of the ascending aorta. (a) Original image, (b) the circle detected by the improved seed detection method based on Hough transformation, (c) the initial seeding for the segment *C*_*m*_ center is marked as the aorta (blue), and the pixels that are 1.5 *C*_*m*_ distance away from the *C*_*m*_ center are marked as background (red). The green line is the border of the aorta segmented by initial seeding. (d) The adaptive seed transfer provided by the segmentation result of the previous slice and the segmented aorta borders.

In the binary geodesic distance segmentation algorithm, the foreground and background segmentation seeding pixels are required to provide the zero geodesic distance points [5, 7]. The minimum weighted path distance to the segmentation seed marks are found for each pixel. The label for a pixel is found by choosing the shorter geodesic distance to the foreground or background markings. The detected aorta circle, *C*_*m*_, provides an initial seed for the 2D geodesic segmentation in slice *m*. The center pixel of *C*_*m*_ is assigned as an aorta seed. The pixels that are 1.5 × *⌀C*_*m*_ distance away from the *C*_*m*_ center are marked as background segmentation seeds. 1.5 × *⌀C*_*m*_ distance away from the *C*_*m*_ center is an arbitrary large distance from the aorta that can be marked as background segmentation seeds for geodesic segmentation. See Figure 1(c). The labels are denoted as *𝒜* and *ℬ* for the ascending aorta and background. The sets of the seeding pixels are denoted as *Ω*_*𝒜*_ and *Ω*_*ℬ*_. The geodesic distances from each pixel to the seed markings are computed as

(2) $$\begin{array}{c} D_{l}\left(\;x\;\right)=\underset{s\in \Omega_{l}}{\min}\;d\left(\;s,x\;\right),\;l\in \left\{A,B\right\}, \end{array}$$

where

(3) $$\begin{array}{c} d\left(\;s_{1},s_{2}\;\right)=\underset{C_{s_{1},s_{2}}}{\min}\overset{1}{\underset{0}{\int }}\nabla I{\left(\;p\;\right)}^{2}C_{s_{1},s_{2}}\left(\;p\;\right)dp. \end{array}$$

*D*_*l*_(*x*) calculates the minimum geodesic distance between the seed pixels and pixel *x*. A pixel is labeled *𝒜* if *D*_*𝒜*_ is shorter than *D*_*ℬ*_; otherwise, the pixel is labelled *ℬ*. In (3), the minimum geodesic path is found between two points. *C*_*s*_1_,*s*_2__ is a path through pixels *s*_1_ and *s*_2_. The path is weighted by the square of the gradient, where *I*(*p*) is the CT intensity. The square of the gradient weight ensures that the minimum geodesic distance is through the low gradient pathway.

To find *D*_*l*_(*x*) for each pixel, the raster-scan algorithm is implemented. The raster-scan algorithm updates the minimum distances for each pixel sequentially over an image in multiple passes [15, 16]. With the optimal complexity of *O*(*N*), where *N* is the number of pixels, the geodesic distance transform can be found almost instantaneously per axial slice.  Figure 1 shows an example of the segmentation process for an axial slice; (a) shows slices of the input data, (b) shows the detected initial seed point by Hough transformation, and (c) shows the segmented aortic borders by the initial seeding using the Hough circle.

### 2.4. Adaptive Seed Transfer

Once the aortic circle is segmented in slice *m*, the adjacent axial slices seeding pixels can be marked, which in turn can be further refined by geodesic distance segmentation. In this subsection, we will refer to an already segmented slice as the reference slice. The reference slices segmentation result is the seed for the segmentation seeding of adjacent axial slices.

Because of the obtuse shape of the ascending aorta, we only expect to find a moderate change of aorta segmentation in the adjacent axial slice. We can safely assume that two adjacent slices largely share the same segmentation labels, except the pixels around segmentation borders, which are less likely to have the same label as the adjacent frames. The border or edge pixels are pixels with different segmentation labels for at least one of the neighboring pixels. The distance *D*_*E*_ to the bordering pixel *s* ∈ *Ω*_*E*_ is updated using same* raster-scan* algorithm:

(4) DEx=mins∈ΩE⁡ ds,x,where  ds1,s2=minCs1,s2⁡∫01Cs1,s2pdp.

The previous sections geodesic distance for segmentation uses the squared gradient as the weight factor. In (4), the weight is simply equal to one, which means that *D*_*E*_(*x*) can be translated as the minimum distance to one of bordering pixels. The* raster-scan* algorithm with an 8-neighbouring pixel structure, however, can deliver a computationally faster approximation of the minimum distance.

*x*_*j*_ is a pixel in the adjacent slice, and *x*_*r*_ is the pixel in the reference slice that shares the same *xy* coordinate points. The adjacent slices pixel *x*_*j*_ is marked as the aorta seed if the reference slice pixels *D*_*E*_(*x*_*r*_) is greater than the threshold and if the reference slices pixel *x*_*r*_, which is located at same position, is segmented as the aorta:

(5) $$\begin{array}{c} x_{j}\in \Omega_{A}\;\text{if }D_{E}\left(x_{r}\right)>T_{A}\wedge x_{r}\in A. \end{array}$$

Otherwise, the adjacent slices pixel is the background seed if *x*_*j*_ is within a distance range from the bordering pixels:

(6) xj∈ΩBif  DExr>Tmin∧DExr<Tmax∧xr∈B.

By transferring the segmentation annotation seeds through the axial slices and performing geodesic segmentation, 3D segmentation of aorta is achieved. Due to the distance threshold during foreground seed transfer, the foreground seed will not transfer once the reference slices foreground becomes small. When no further aorta seed transfers are made, the cross axial slice segmentation process stops.

## 3. Experimental Results

For all of the datasets, the proposed method provided a successful segmentation of the ascending aorta regardless of the image quality. The ascending aortic circles for initializing the segmentation were successfully detected by the proposed multiple Hough circle detection method. In this process, the aortic circles were almost consistently obtained over the axial slice, except for some nonsuccessive slices, which accidentally include the circular structures that have similar intensities and sizes as the aorta in axial direction. However, the proposed Hough circle selection method could overcome this problem and detect the correctly placed aorta circle. After detection of the aortic circle, simple initial segmentation seeds are designated and the VOIs were also set up across all of the axial slices that contain the ascending aorta. Then, the aorta segmentation started from initial reference slice and the aorta was segmented repeatedly up and down the slice in the VOI by the geodesic distance algorithm until the aortic arch or valve was reached through adaptive seeding from the adjacent slice. Finally, the ascending aorta was successfully segmented and separated from the left ventricle for all of the datasets.

**Figure 2:**
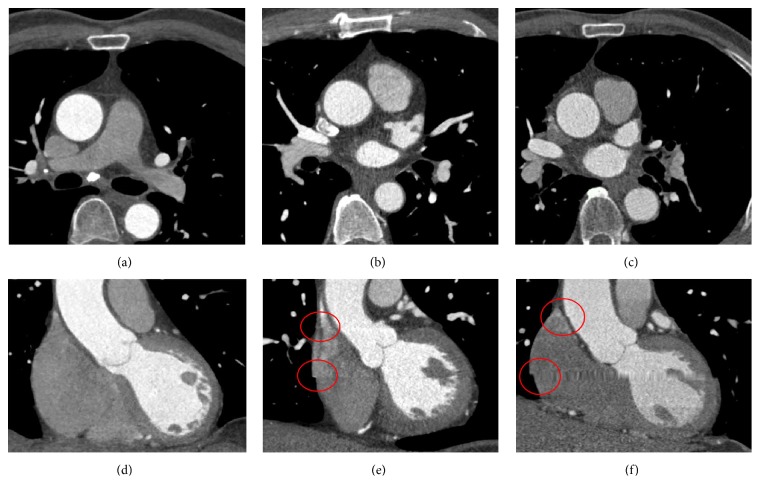
The typical good, moderate, and poor CCTA images used in the evaluation. These images were graded according to noise and artifacts such as step artifacts due to wrong registration and beam hardening. (a) and (d) show images of good quality with little noise and no step artifact (data#000). (b) and (e) show images of moderate quality with some noise and minor step artifact (data#003). (c) and (f) show images of poor quality with noise and major step artifact (data#008). The images on the top are axial plane ((a),(b),(c)) and the images at the bottom are coronal plane((d),(e),(f)).

We evaluated the proposed method by comparing the results with ground truths and a commercially available workstation (Vitrea, Vital Images) [17]. The test data set consists of 10 CCTA images, where there were 3 good, 4 moderate, and 3 poor images, which were graded by a cardiologist with five years of experience.  Figure 2 shows the typical good, moderate, and poor CCTA images. These *X*-*Y* dimensions are 512 × 512 pixels, with a pixel size of 0.38 mm, and the number of slices of all of the datasets varies between 206 and 275 slices, with slice thickness between 0.3 and 0.8 mm. The ground truth was manually annotated by human experts and the approved and experienced cardiologist to produce the correct segmentation of each dataset to evaluate our results.

The Dice Similarity Coefficient (DSC) was used to compare the results of the proposed method *S*_*A*_ to the ground truth *S*_GT_ and those of the commercial workstation *S*_*C*_. *S*_*A*_ and *S*_GT_ are the set of pixels in the 3D scan that have labels that belong to the aorta. The DSC is defined as 2|*S*_GT_∩*S*_*A*_|/(|*S*_GT_ | +|*S*_*A*_|), where 0 signifies the zero overlap between the ground truth segmentation and the derived segmentation result and 1 signifies the complete overlap between ground truth and segmentation result in both the foreground and background.

The proposed method and commercial workstation produced the DSC values, as shown in Table 1, compared with the manually segmented ground truth. In the proposed method, the mean value of DSC with the ground truth was 0.9498, the standard deviation was 0.01548, the maximum value was 0.9714, and the minimum value was 0.9230. According to the commercial workstation, the mean value of DSC was 0.9072, the standard deviation was 0.0140, the maximum value was 0.9313, and the minimum value was 0.8873.

The computation time was measured on a Pentium 4 computer with 3.50 GHz and 16 GB of RAM. The mean computation time for the ascending aorta segmentation was 1.5188 sec, the standard deviation was 0.4157 sec, the maximum value was 1.9670 sec, and the minimum value was 0.9310 sec. The commercial workstation was able to segment an ascending aorta for all of the dataset within approximately 7 seconds. The segmentation time for each piece of data was obtained by averaging 3 separate measurements obtained by stop-watch. However, we cannot accurately measure the computation time for the commercial workstation. The commercial workstation automatically separates heart region from a CCTA image immediately after a CCTA image is loaded into memory which we cannot measure without knowing the source codes. Then, the following ascending aorta segmentation can be measured with stop-watch. Although the computation time can be shown to be more efficient for the proposed method, we compare our results and those of commercial workstation with a focus on the accuracy of segmentation.

**Figure 3:**
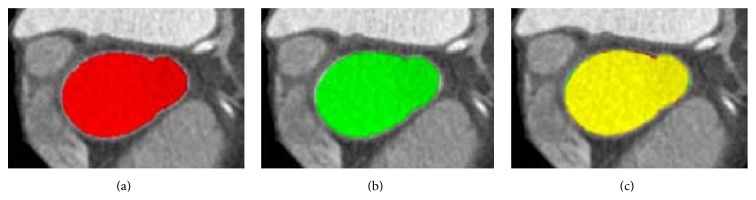
Comparison of the segmentation result with the ground truth. (a) The manually segmented ground truth (red). (b) The result of automatic segmentation by the proposed method (green). (c) The overlapped ground truth and the segmentation result. The overlapped pixels are expressed as yellow.

**Figure 4:**
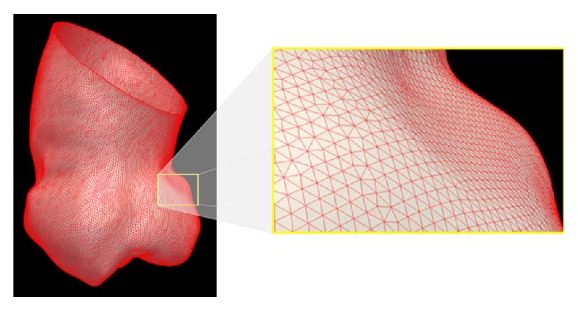
Visualization of the three-dimensional triangular mesh made from the segmented aorta using the Marching Cube algorithm. The whole mesh of the segmentation result and a close-up of the whole mesh.

Figure 3 shows the segmented results. In Figure 3, (a) is the manually segmented ground truth (red), (b) is the result of the presented automatic method (green), and (c) is the overlap of the manual and automatic segmentation (yellow). After segmentation, the mesh model of the ascending aorta is finally generated. The mesh model of the segmented ascending aorta is shown in Figure 4.

## 4. Discussion

All of the DSC values from the proposed method have a DSC value greater than 0.92, and the accuracy of proposed method was higher than that of the commercial workstation for all of the test datasets. The commercial workstation showed slightly overestimated results, which agreed with the results of the work of Oberoi et al. [18]. However, there are still problems with FPs (false positives) and FNs (false negatives), although proposed method showed good accuracy compared to commercial workstation. The most segmentation errors occurred at the end of aorta around three aorta leaflets. In some cases, either a slight leakage of left ventricle was observed, which is due to the unclear border between aortic valve and left ventricle, or the adaptive seeding method tended to terminate early and aortic valve regions were unsegmented.

The proposed method showed high performance for computation time. The average computation time was 1.5188 sec. All of the datasets were segmented within 2 seconds. The computation time was slightly dependent on the FOV (field of view) as well as the number of slices containing the ascending aorta; however, the differences are barely notable.

## 5. Conclusions

In this paper, we developed a method for the fast and fully automatic extraction of the ascending aorta from CCTA images. The method incorporates two separated algorithms, a circular Hough transformation for initialization and the Geodesic distance algorithm for segmentation. The circular Hough transformation algorithm could automatically find the most probable ascending aortic circle in the axial image. The initial seeding was found, and the VOI was defined after detecting the aortic circle. Then, the boundary of the ascending aorta was repeatedly delineated slice by slice by the geodesic distance algorithm until the aortic arch or valve was reached or until the ascending aorta was completely segmented. The proposed method was evaluated and compared with the results from other commercial workstations using 10 data sets. For all of the datasets, the proposed method segmented the ascending aorta successfully. The proposed method showed a highly accurate DSC with ground truth, and high performance regarding the computation time was measured. Future work will focus on the segmentation of the aortic valve to overcome the aforementioned limitations mentioned in Section 4.

## Figures and Tables

**Table 1:** DSC values to compare accuracy of the proposed method and computation time.

Data#	Quality	DSC		Number of slices	Computation time (sec)
		Proposed method	Workstation		
000	Good	0.9714	0.9092	227	1.8820
001	Good	0.9500	0.8917	219	0.9310
002	Good	0.9511	0.8873	213	1.1150
003	Moderate	0.9660	0.9226	243	1.9100
004	Moderate	0.9548	0.9091	206	1.2480
005	Moderate	0.9397	0.8935	247	1.8140
006	Moderate	0.9230	0.9047	275	1.9670
007	Poor	0.9493	0.9313	269	1.8170
008	Poor	0.9628	0.9178	245	1.5430
009	Poor	0.9295	0.9045	134	0.9610

Average		0.9498	0.9072	228	1.5188
